# Simulations of Novel Semi-Spherical Electrode Detectors Formed by Simultaneously Deep-Etched Trenches

**DOI:** 10.3390/mi17050627

**Published:** 2026-05-20

**Authors:** Hongfei Wang, Zheng Li

**Affiliations:** 1 School of Physics and Physical Engineering, Qufu Normal University, Qufu 273165, China; wanghf.20@163.com; 2 School of Integrated Circuits, Ludong University, Yantai 264025, China; 3 Engineering Research Center of Photodetector Special Chip in Universities of Shandong, Ludong University, Yantai 264025, China

**Keywords:** DRIE Uneven-Etched 3D-Spherical electrode detector, DRIE etching method, electric potential distributions, electric field distribution, electron concentration distribution, full depletion voltage, leakage current, capacitance

## Abstract

A novel 3D detector with a semi-spherical electrode detector structure is proposed in this study. The semi-spherical electrode is formed by concentric deep circular-type trenches of varying depths. These concentric trenches can be simultaneously deep-etched using DRIE (Deep Reactive-Ion Etching) depths obtained from our calculations for a certain time at a given aspect ratio. The focus of this work is the conceptualization, design considerations, 3D modeling, and electrical simulation of the proposed 3D detector. The detector’s electrical properties, including electric potential distribution, electric field distribution, electron concentration distribution, full depletion voltage, leakage current, and capacitance, were simulated using a technology computer-aided design (TCAD) tool. Simulation and analysis of the detector’s performance post-irradiation were also conducted. The small capacitance of our semi-spherical electrode detector renders it highly suitable for applications in photon sciences (e.g., X-ray).

## 1. Introduction

**Figure 1:**
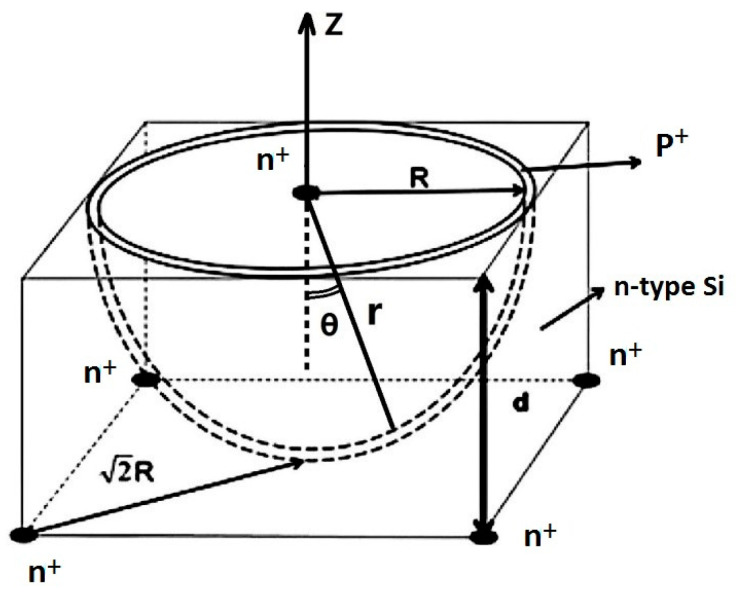
The hypothetical sphere-electrode detector.

Recently, owing to the advancement of three-dimensional (3D) electrodes, specifically column electrodes [1,2,3,4,5,6,7,8,9], as well as detectors based on 3D-trench electrodes [10,11,12,13,14,15], a hypothetical sphere-electrode detector, shown in Figure 1, has been modeled and simulated [16]. Modeling and simulation [17] provide a solid theoretical foundation for the implementation of practical near-semi-sphere and semi-sphere-electrode detectors. In recent years, theoretical research aiming to realize 3D-Spherical electrode silicon detectors through laser drilling [18] and epitaxy [19] methods has been conducted. Our new detector design and processing technology are different from those in ref. [18], where separate drillings are used for each trench, with each trench having a different depth. In our case, trenches with different depths are etched simultaneously (one etch), which is a time-saving process. In addition, the trench walls are passivated by the RIE process with fewer defects.

Parker et al. proposed the 3D electrode detector [1]. Despite material and structural advancements in numerous semiconductor detectors, silicon detectors retain unique benefits, including high resolution, rapid response speed, and straightforward integration [20]. Silicon detectors are also versatile and used in numerous areas, such as X-ray imaging, high-energy physics, photon sciences, and aerospace [21,22,23,24]. In the 3D silicon detector architecture, electrodes are embedded within the silicon material. Compared with the two-dimensional (2D) electrode detector structure, 3D silicon detector electrodes are embedded into the silicon substrate, which makes the full depletion voltage independent of the thickness of the silicon bulk, but only in relation to the electrode spacing [25]. In practice, p-type bulk material is selected for the 3D-trench electrode detector to prevent space charge sign inversion, while n-type silicon bulk is typically used in photon science [26].

The DRIE Uneven-Etched detector offers two key advantages compared to traditional 3D electrode detectors: (1) its charge collection efficiency remains largely independent of θ; (2) it exhibits more symmetrical electrical characteristics including electric potential distribution, electric field distribution, and electron concentration distribution; and it has a low full depletion voltage and small capacitance. This study will explain the advantages of the proposed detector through structural optimization and technology computer-aided design (TCAD) simulations. Modeling and simulation establish a solid theoretical foundation to guide subsequent fabrication processes of the Uneven-Etched 3D-Spherical electrode detector using the DRIE etching method. Since DRIE for deep trenches is a relatively mature technology in Europe (e.g., CNM of Spain [7,26], FBK of Italy [3], and IMCAS in China [17]), the fabrication of the proposed detector is believed to be feasible and achievable, and therefore practical.

## 2. The DRIE Uneven-Etched 3D-Spherical Electrode Detector Concept and Design Considerations

**Figure 2:**
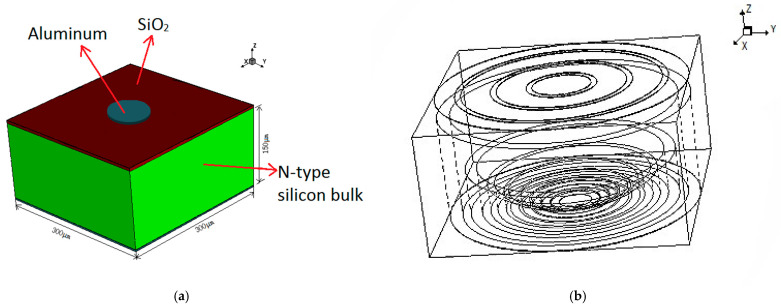
(**a**) Simulated 3D structure of the DRIE Uneven-Etched 3D-Spherical electrode detector cell. (**b**) A perspective drawing.

**Figure 3:**
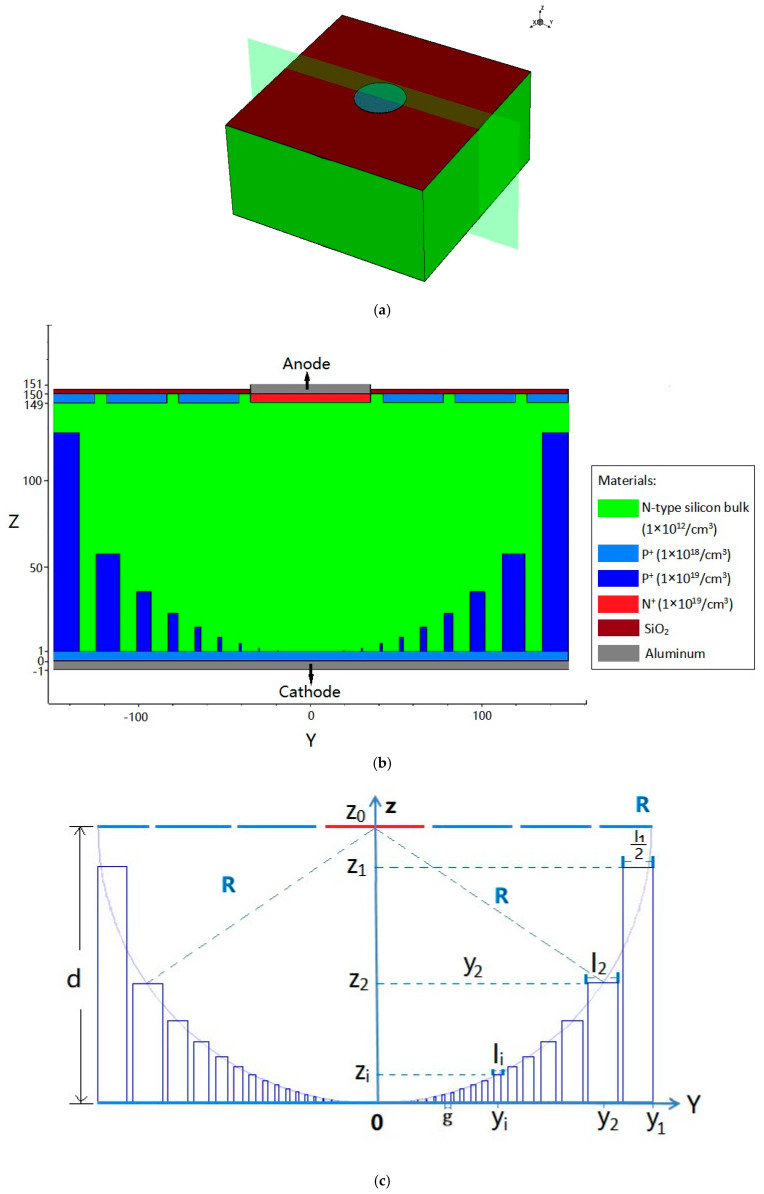
(**a**) The unit with a 2D cross-section cut at X = 0. (**b**) Sectional view. (**c**) Structural parameters of the 3D-Spherical cathode.

As shown in Figure 2 and Figure 3, the detector unit is 150 μm thick with a square configuration of 300 × 300 μm^2^. The anode is situated on the cell’s top layer as a centrally placed, circular doped electrode measuring 70 μm in diameter. The anode is n+-type doping with a concentration of 1 × 10^18^ cm^−3^ and a doping depth of 1 μm. The anode electrode, surrounded by three floating rings with the same gap and the same width, is located in the top-layer center of the unit cell. The gap and the width of the three floating rings are 7 μm and 35 μm, respectively. All three floating rings are p+-doped, the doping concentrations are 1 × 10^18^ cm^−3^, and the doping depths are 1 μm. The n-type silicon bulk has a doping concentration of 1 × 10^12^ cm^−3^. At the bottom of the silicon bulk, there are nine circular trenches nested with each other. The nine circular trenches, which are p+-doped, have the same ratio of height to width, and the doping concentrations are 1 × 10^19^ cm^−3^. A p+ implant at the bottom of the silicon substrate connected these nine circular trenches together; the doping concentration is 1 × 10^18^ cm^−3^, while the whole doping layer has a depth of 1 μm. The cathode electrode, composed of these nine circular trenches and the whole bottom layer, is the cathode electrode of the 3D-Spherical electrode detector. Figure 3 shows that the radius of the 3D-Spherical electrode detector is 150 μm. An aluminum electrode contact layer, measuring 1 μm in thickness, covers the entire bottom surface. The anode electrode on the cell’s top surface is conformally coated with a 1 μm thick aluminum contact layer. The exposed regions of the silicon bulk are subsequently passivated with a 0.5 μm thick SiO_2_ layer.

Figure 3c is a 2D cross-sectional view at X = 0 μm that shows the simultaneously etched trenches of the detector. The structural parameters of the 3D-Spherical cathode and the design rules of the detector concept can be clearly seen: z_i_ and l_i_ represent the depth and the width of the etched circular trench, respectively, and the variable d denotes both the detector’s thickness and the vertical separation between the centers of the cathode and anode. The radius of the 3D-Spherical cathode is denoted by R. An experiment is carried out to find the maximum etching time (T) needed to etch through the entire wafer thickness (d = R) at a given trench width l_0_ (e.g., l_0_ = 35.29 μm). Subsequently, this T value is used for the simultaneous etching of trenches, and because the etching time (T) is the same, z_i_ is proportional to l_i_ (the ratio of etching height z_i_ to etching width l_i_ is the same, i.e., aspect ratio B). In Figure 3c, y_i_ is the *Y*-axis coordinate in the middle of the circular trench, and g is the gap between the two adjacent circular trenches. These parameters above, which meet the requirements for Equations (1)–(9) below, are needed to jointly construct the 3D-Spherical electrode. We can obtain 3D-Spherical electrode detectors in different sizes by varying these structural parameters. Figure 3 shows the structure (z_0_ = R = 150 μm; l_1_ = 30 μm; g = 10 μm).

Given d = R, y_1_ = R, z_0_ = R, z_1_ = βR (0 < β < 1), l_0_ = 35.29 μm, β = 0.85, and g = g_0_ = constant, we have

(1) $$\frac{z_{i}}{l_{i}}=\frac{z_{1}}{l_{1}}=\frac{\beta R}{l_{1}}=\mathrm{constant}=B=\frac{R}{l_{0}}$$

B can be obtained from the experiment stated above (i = 1, 2, …, N):

(2) $$\left\{\begin{array}{c} l_{2}=l_{1}\frac{z_{2}}{z_{1}}=\frac{z_{2}}{B}\\ z_{2}=R-\sqrt{R^{2}-y_{2}^{2}}\\ y_{2}=y_{1}-\frac{1}{2}l_{2}-g-\frac{1}{2}l_{1} \end{array}\right.\;C_{1}=y_{1}-g-\frac{1}{2}l_{1}\;z_{2}=\frac{\left(\frac{C_{1}}{B}\;+2R\right)-\sqrt{{\left(\frac{C_{1}}{B}\;+2R\right)}^{2}-4\left(\frac{1}{4B^{2}}\;+1\right)C_{1}^{2}}}{2\left(\frac{1}{4B^{2}}\;+1\right)}$$

Therefore, we obtain z_2_ = 57.668 μm, l_2_ = 13.569 μm, and y_2_ = 118.215 μm.

(3) $$C_{i}=y_{i}-g-\frac{1}{2}l_{i}\;z_{i+1}=\frac{\left(\frac{C_{i}}{B}+2R\right)-\sqrt{{\left(\frac{C_{i}}{B}+2R\right)}^{2}-4\left(\frac{1}{4B^{2}}+1\right)C_{i}^{2}}}{2\left(\frac{1}{4B^{2}}+1\right)}$$

(4) $$z_{i+1}=R-\sqrt{R^{2}-y_{i+1}^{2}}$$

(5) $$y_{i+1}=y_{i}-\frac{1}{2}l_{i+1}-g-\frac{1}{2}l_{i}$$

(6) $$l_{i+1}=\frac{z_{i+1}}{B}$$

From Equation (4), we have

(7) $$R^{2}-y_{i+1}^{2}={\left(R-z_{i+1}\right)}^{2}$$

From Equations (6) and (7), we obtain

(8) $$R^{2}-y_{i+1}^{2}={\left(R-{\mathrm{Bl}}_{i+1}\right)}^{2}$$

From Equation (5), we have

(9) $$l_{i+1}=2y_{i}-2y_{i+1}-2{\mathrm{gl}}_{i}$$

From Equations (8) and (9), we can write

(10) $$R^{2}-y_{i+1}^{2}={\left[R-B\left(2y_{i}-2y_{i+1}-2g-l_{i}\right)\right]}^{2}$$

or

(11) $$R^{2}-y_{i+1}^{2}={\left[R+2B\left(-y_{i}+g+\frac{1}{2}l_{i}\right)+2{\mathrm{By}}_{i+1}\right]}^{2}$$

If we let

$$A_{i}=R+2B\left(-y_{i}+g+\frac{1}{2}l_{i}\right)$$

then we obtain

(12) $$R^{2}-y_{i+1}^{2}={\left[A_{i}+2{\mathrm{By}}_{i+1}\right]}^{2}$$

Finally, we obtain the equation for y_i+1_ as a function of y_i_, l_i_:

(13) $$\left(4B^{2}+1\right)y_{i+1}^{2}+4{\mathrm{BA}}_{i}y_{i+1}+\left(A_{i}^{2}-R^{2}\right)=0$$

(14) $$y_{i+1}=\frac{-4{\mathrm{BA}}_{i}+\sqrt{{\left(4{\mathrm{BA}}_{i}\right)}^{2}-4\left(4B^{2}+1\right)\left(A_{i}^{2}-R^{2}\right)}}{2\left(4B^{2}+1\right)}$$

Then, we can obtain all l_i_, y_i_, and z_i_ values.

The simultaneous etching of trenches can be carried out as follows:

(1) An experiment is required to find the maximum etching time (T) needed to etch through the entire wafer thickness (d = R) at a given trench width l_0_ (e.g., l_0_ = 35.29 μm). This T value can be used for the simultaneous etching of trenches.
(2) The circular trenches are designed with widths (l_i_, i = 1, 2,⋯N) from the calculated results of Equations (1)–(14).
(3) Photolithography is carried out on the backside of the detector to define the circular trenches.
(4) DRIE simultaneous etching of trenches is achieved using time T found in (1).
(5) As a result, a semi-spherical electrode at the bottom of the wafer is achieved.
(6) The etched trenches are doped via diffusion of boron to define the p+ semi-spherical electrode.
(7) Photolithography and ion implantation are used to obtain a circular n+ collection anode to form the semi-spherical electrode detector.

Notably, in a real detector process with DRIE, aspect ratio B (which can be higher than 20:1) is obtained from the overall batch process of the Si etch–side-wall protection–Si etch cycle [27]. In our work, the optimistic (or simplified) method is used for the purpose of demonstration. In real applications, however, one may need to modify the method to offset the deviation from the ideal situation, e.g., dividing trench etching into two or more batches with different times (instead of just one etching process in this case) to achieve more accurate results. Furthermore, in real trench electrode processing, diffusion of phosphorous (for n+ electrode doping) and boron (for p+ electrode doping), as well as trench filling of poly-silicon, is needed for better mechanical strength, which will be discussed in future work of detector fabrication.

## 3. Electrical Characteristic Results

### 3.1. Electric Potential Distribution of the DRIE Uneven-Etched 3D-Spherical Electrode Detector

Our simulation tool, known as Sentaurus Device, is an important component of the TCAD tool suite. It is widely used to simulate and analyze the electronic behavior and physical characteristics of various semiconductor devices. Its command file consists of several sections, including File, Electrode, Thermode, Physics, Plot, Math, and Solve, each performing specific functions. The tool can simulate one-dimensional, two-dimensional, and three-dimensional geometries and employs physically based mathematical models to simulate device performance. These models mainly include generation–recombination models [28], mobility models, and tunneling models. The fundamental physical equations include Poisson’s equation, current continuity equations, and carrier transport equations, which are used to calculate performance parameters such as electron transport, electrical characteristics, and carrier concentration in semiconductor devices. It supports the simulation of a wide variety of semiconductor devices, such as MOSFETs, BJTs, diodes, solar cells, and sensors. During simulation, users can customize input/output, bias voltages, physical models, and other conditions to debug semiconductor device performance and for deeper research purposes.

To visualize the electric potential and electric field distribution more clearly within the detector, we defined a 2D cross-section obtained at X = 0 μm in the unit shown in Figure 3a.

**Figure 4:**
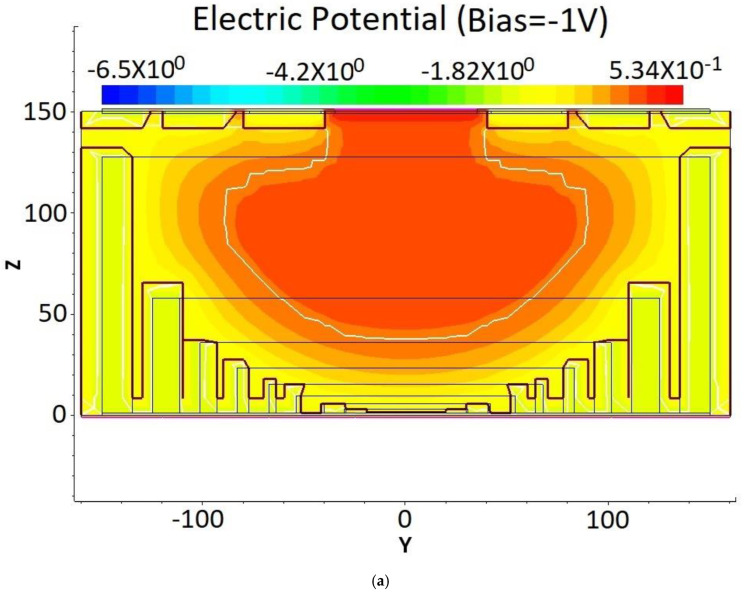

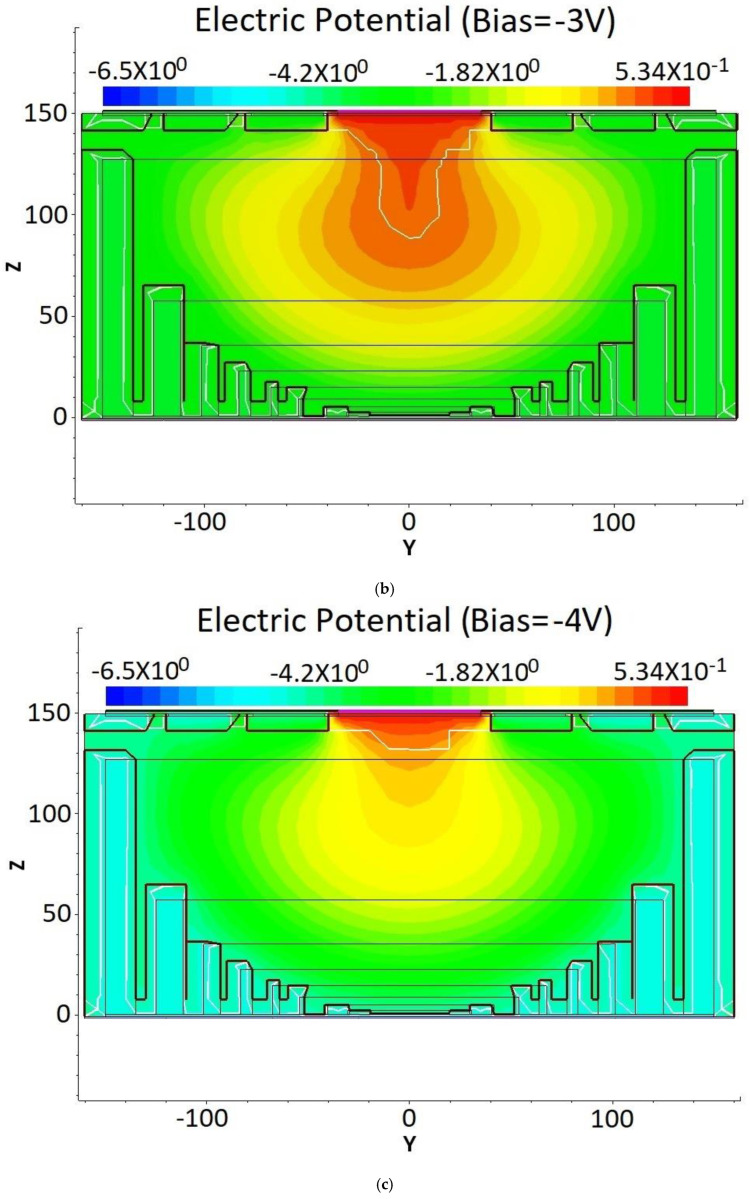

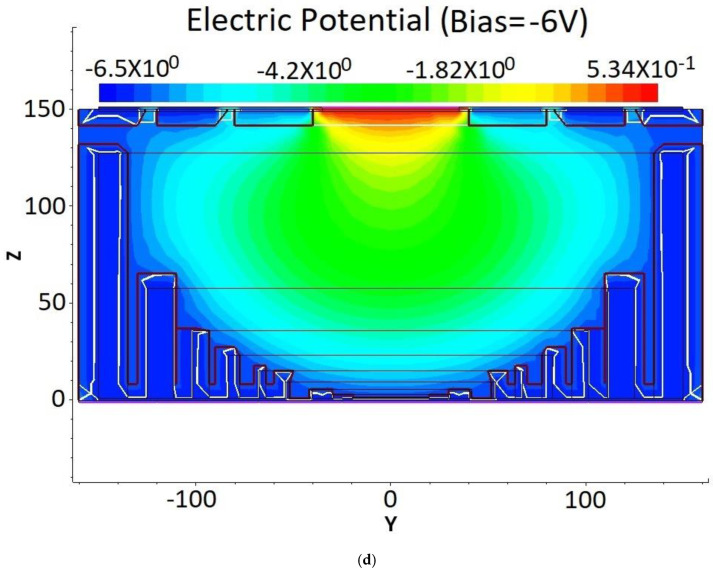
Electric potential profile of the DRIE Uneven-Etched 3D-Spherical electrode detector, biased at (**a**) −1 V, (**b**) −3 V, (**c**) −4 V, and (**d**) −6 V.

**Figure 5:**
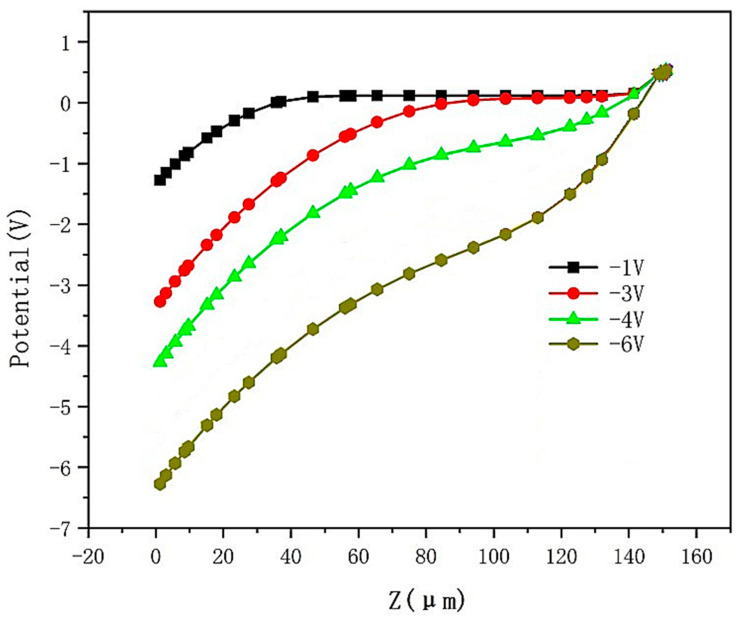
Electric potential profiles of the DRIE Uneven-Etched 3D-Spherical electrode detector at a cut of Y = 0, as shown in Figure 4.

The simulated electric potential distribution of the DRIE Uneven-Etched 3D-Spherical electrode silicon detector unit is depicted in Figure 4. Evidently, a highly uniform and symmetrical potential distribution can be observed. As the bias voltage goes from −1 V to −6 V, the electric potential distribution maintains near-spherical symmetry, consistent with the detector structure and our expectations. Figure 5 shows the detector’s internal potential profiles for bias voltages of −1 V, −3 V, −4 V, and −6 V, as measured along the Y = 0 cross-section in Figure 4 (cut at Y = 0). These curves show that at a bias of −1 V, the electric potential is flat (zero gradient or zero electric field) for Z > 50 μm, indicating that the detector is not yet fully depleted in the region of 50 μm < Z ≤ 150 μm. This non-fully depleted region shortens to 100 μm < Z < 150 μm at a bias of −3 V. At a bias voltage of −4 V, this flat electric potential region disappears, indicating that the detector is nearly fully depleted. It is clear that at a bias of −6 V, the electric potential gradient is non-zero in the entire detector and near-linear, indicating full depletion and a near-constant electric field in the detector.

### 3.2. Electric Field Distribution for the DRIE Uneven-Etched 3D-Spherical Electrode Detector

**Figure 6:**
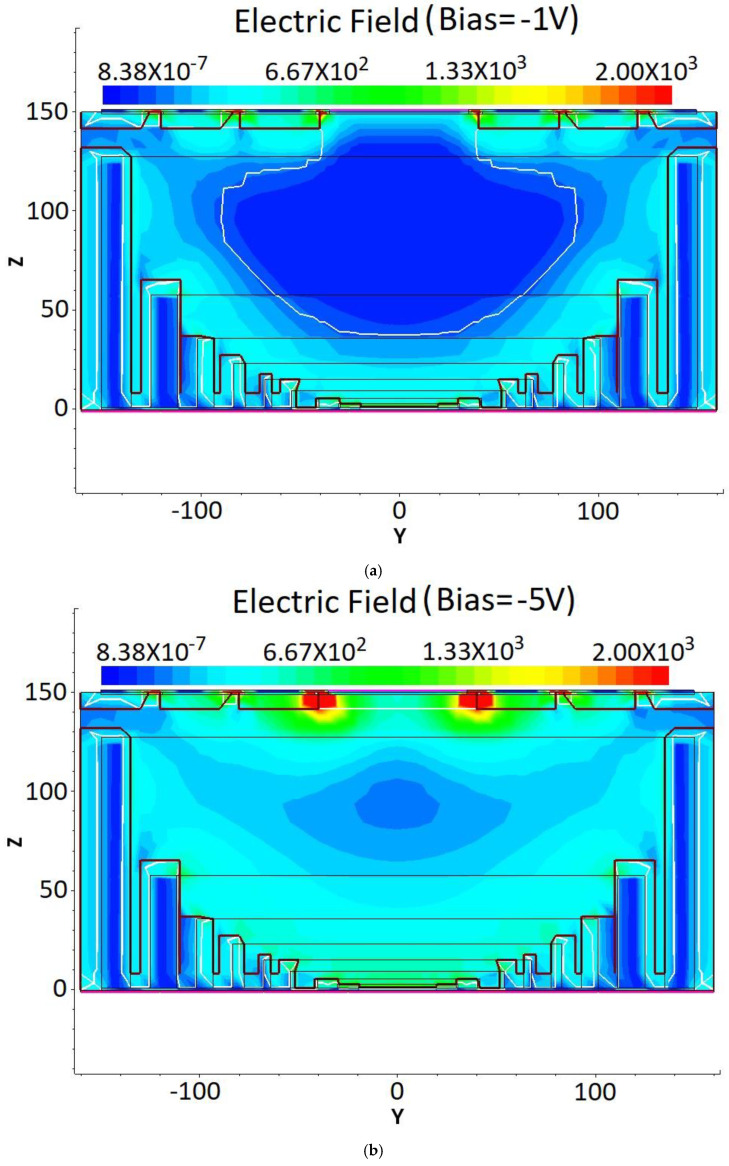

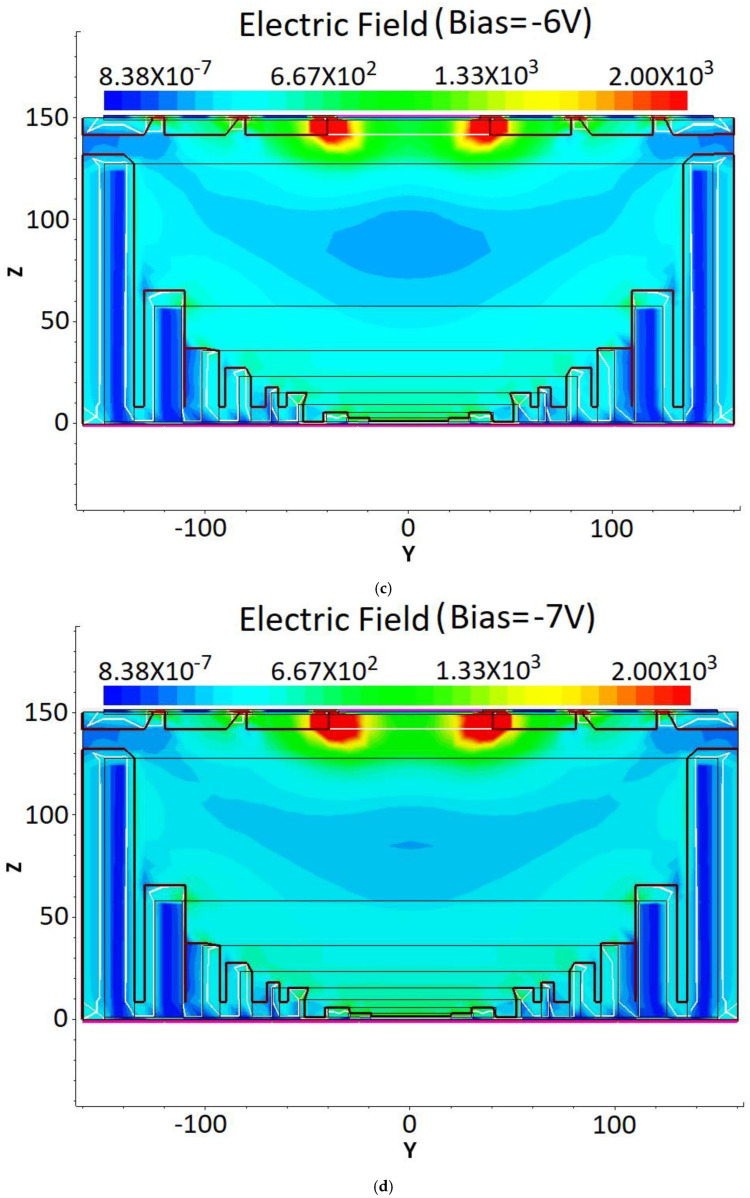

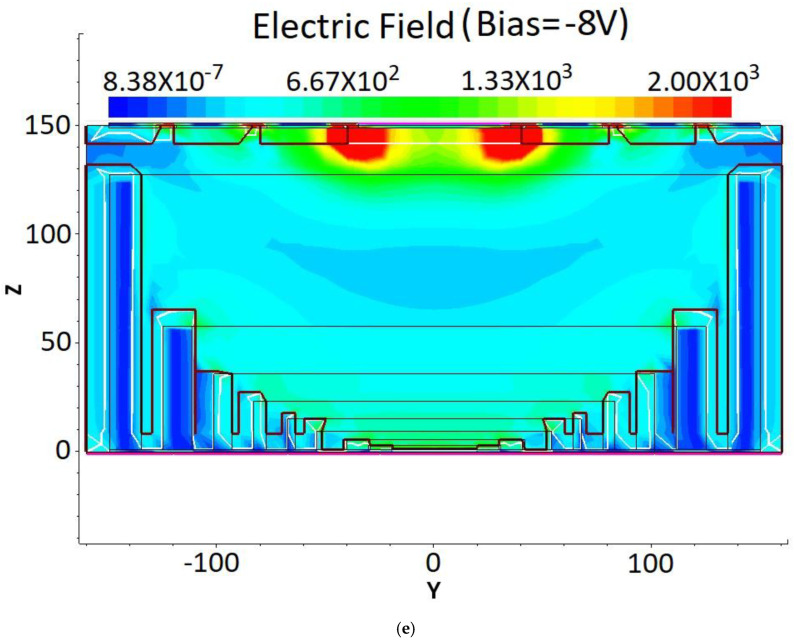
Two-dimensional electric field distributions of the DRIE Uneven-Etched 3D-Spherical electrode detector biased at (**a**) −1 V, (**b**) −5 V, (**c**) −6 V, (**d**) −7 V, and (**e**) −8 V.

Figure 6 displays the detector electric field distribution in a 2D cross-section obtained at X = 0 μm in the unit shown in Figure 3a for biases from −1 V to −8 V.

From Figure 6, it is clear that the electric field extends from the semi-spherical electrode (cathode) towards the center anode with near-spherical symmetry. At a bias of −1 V, there is a larger region near the center anode with zero electric field, while at a bias of −5 V, this center no-field region is vastly reduced, becoming a low-field region with an electric field strength of <100 V/cm. At a bias of −6 V, this low-field region further reduces, with a value of about 150 V/cm. At biases greater than or equal to −7 V, the center low-field region almost disappears entirely, and the lowest field is >300 V/cm. Full depletion is therefore achieved at around −6 V, which we refer to as the detector full depletion voltage (V_fd_). A radial field configuration is established between the central top anode and the spherical cathode, with all field lines originating at the former and terminating at the latter. Electrons induced by the incident particles drift toward the central collecting electrode as a result of the established electric field distribution.

**Figure 7:**
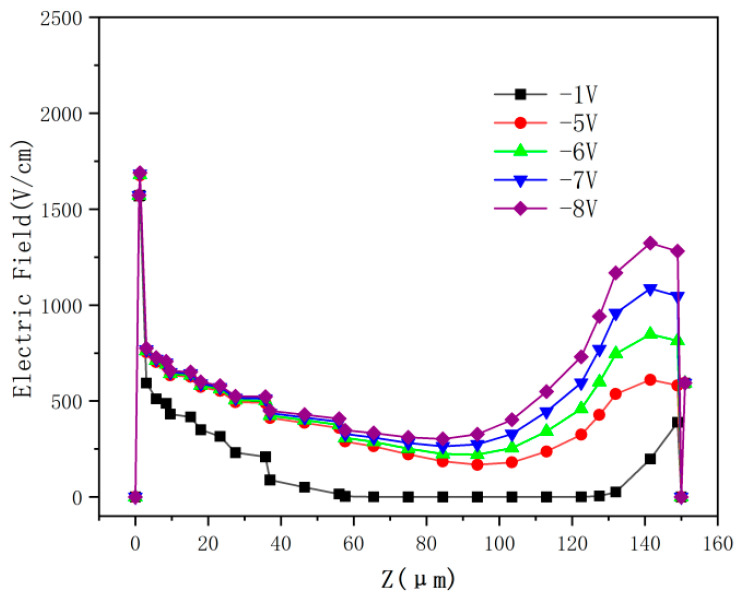
Electric field profile of the DRIE Uneven-Etched 3D-Spherical electrode detector biased at −1, −5, −6, −7, and −8 V.

To further analyze the distribution of the electric field, we plot the curves in a cut of Y = 0 in Figure 6 under different bias voltages, and the results are shown in Figure 7. At a bias voltage of −1 V, the electric field remains 0 V/cm for a large part of the detector. As the bias voltage increases, the electric field progressively extends to the entire detector. At a bias of −6 V, the electric field strength near the top of the detector is approximately 800 V/cm, while that at the bottom of the cathode is 760 V/cm. The minimum electric field at the center of the detector is about 250 V/cm. The average electric field is about 500 V/cm (at −6 V). For electrons drifting from the middle (at Z = 75 μm), the drift time is about 10 ns, which is small enough for most applications. If a faster drift time is needed, one can increase the bias voltage accordingly. Semi-spherical electrode detectors have much more uniform electric field distributions than other types of 3D detectors, and in [18,25,29], further details of the physical analyses of this type of detector are given.

### 3.3. Full Depletion Voltage of the DRIE Uneven-Etched 3D-Spherical Electrode Detector

The calculation formula of the depletion voltage for the detector is as follows [16]:

$$V_{\mathrm{fd}}=\frac{{\mathrm{qN}}_{\mathrm{eff}}d^{2}}{6\varepsilon_{0}\varepsilon }$$

Notably, the full depletion voltage V_fd_ takes the absolute value of bias voltage V.

For the DRIE Uneven-Etched 3D-Spherical electrode detector, phosphorus is incorporated into the silicon bulk as an n-type dopant with a density of 1 × 10^12^ cm^−3^, which defines the net effective doping level (N_eff_ = 1 × 10^12^ cm^−3^). The thickness of the silicon bulk, denoted as d, is 150 μm. Derivation from the depletion voltage equation gives a V_fd_ of 5.67 V. Consequently, the depletion voltage in a practical implementation of the innovative detector is anticipated to be on the order of −6 V.

**Figure 8:**
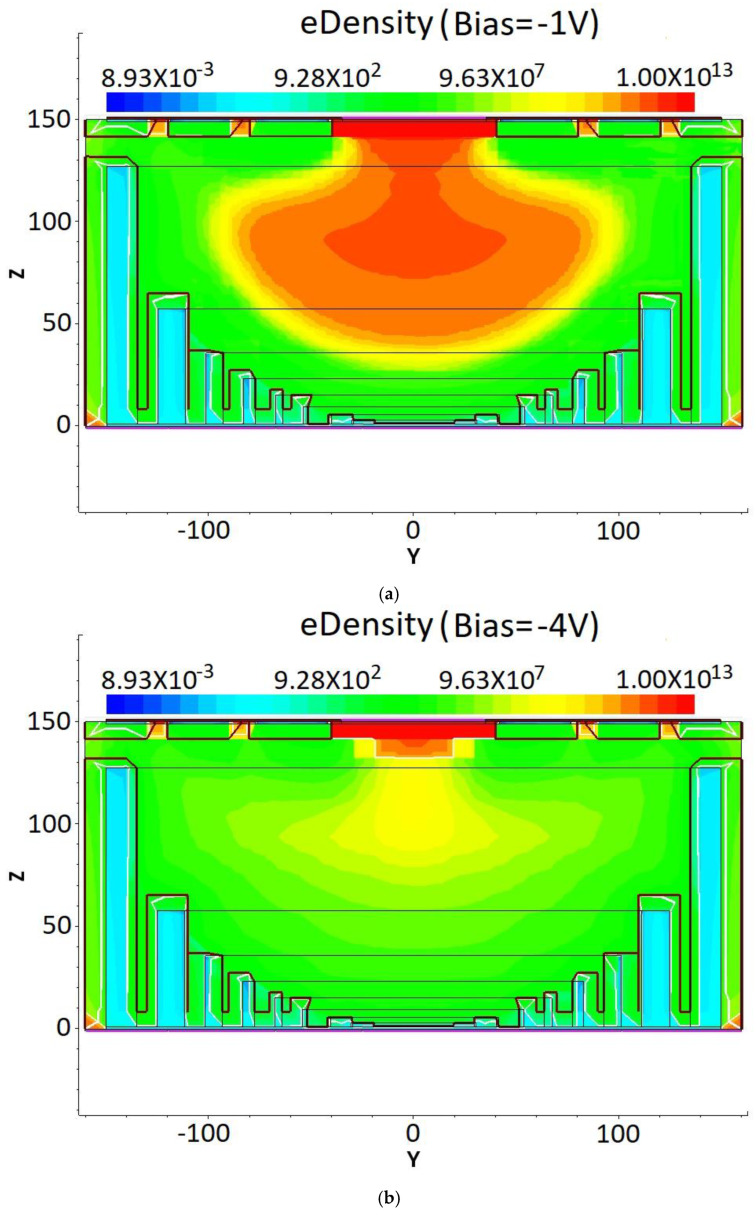

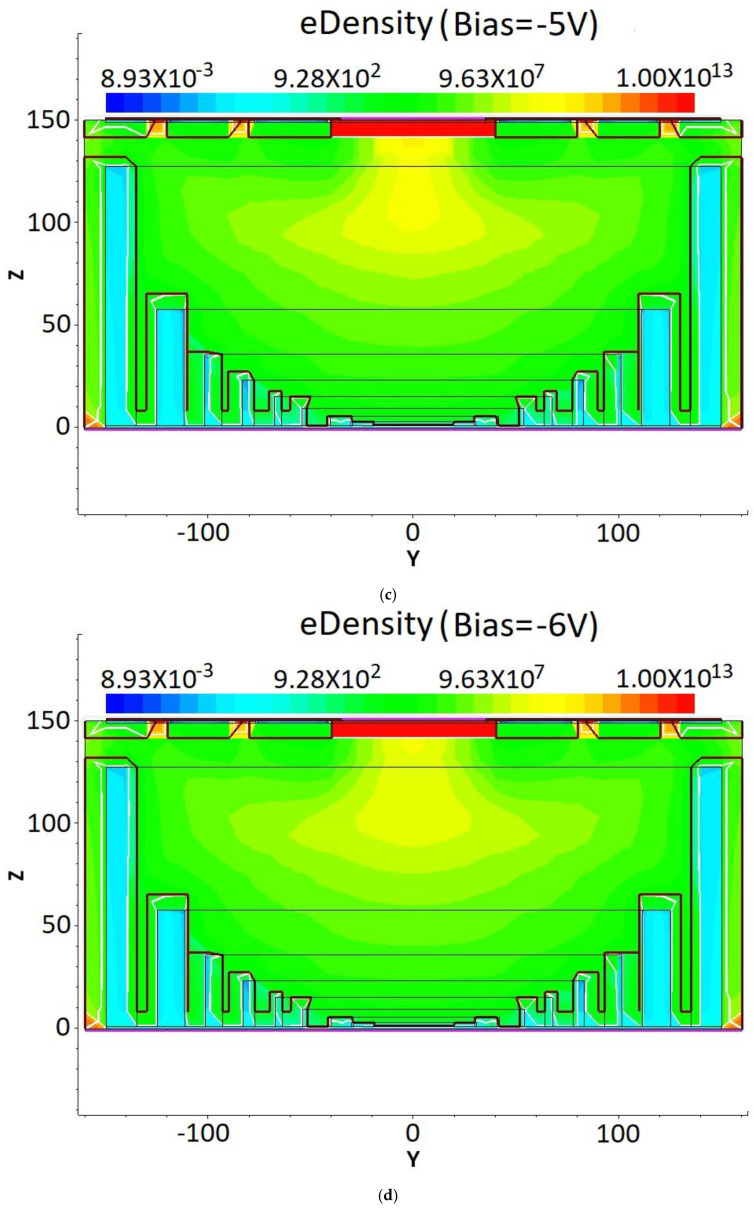
Electron concentration profile for the DRIE Uneven-Etched 3D-Spherical electrode silicon detector biased at (**a**) −1 V, (**b**) −4 V, (**c**) −5 V, and (**d**) −6 V.

**Figure 9:**
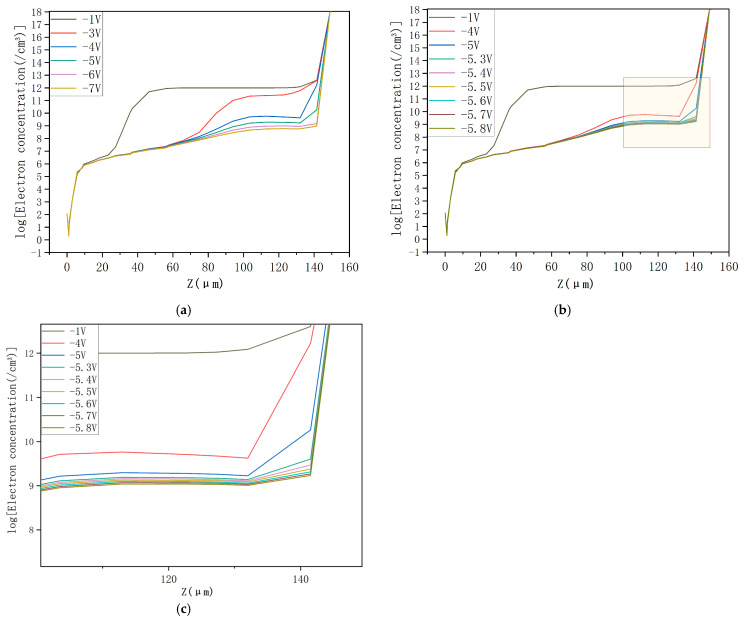
(**a**) Electron concentration and the depth profile of the innovative detector under various voltages. (**b**) Electron concentration in the novel detector under various voltages. (**c**) Close-up view of the concentration depth profile from Figure 9b.

The electron concentration profiles for the DRIE Uneven-Etched 3D-Spherical electrode silicon detector under bias voltages of −1 V, −4 V, −5 V, and −6V are depicted in Figure 8. At a bias of −1 V, the vast part of the detector’s center is non-depleted (Figure 8a). As bias increases, progressive expansion of the depletion region is clearly seen in Figure 8b–d. At a bias of −6 V, the detector is clearly fully depleted. Again, we observe a clear spherical symmetry in the electron concentration profile. Figure 9a illustrates the electron concentration distribution at the cross-section corresponding to Y = 0 in Figure 8, as observed under varying voltages. At a bias voltage of −6 V, the detector is fully depleted of free electrons. It is hypothesized that the depletion voltage for the new detector falls within the range of −5 V to −6 V. To accurately determine the depletion voltage, we modeled the electron concentration in the detector using fine voltage increments from −5 V to −5.8 V, with results shown in Figure 9b. The close-up diagram in Figure 9c, which corresponds to the yellow section in Figure 9b, reveals that the detector is fully depleted of free electrons at an applied bias of −5.7 V. This confirms that the full depletion voltage of our detector is 5.7 V. Within the theoretical and simulation software error allowance, the simulation result corresponds well to the theoretical value of −5.67 V from the depletion voltage equation.

### 3.4. The Leakage Current and the Transient Induced Current of the DRIE Uneven-Etched 3D-Spherical Electrode Detector

**Figure 10:**
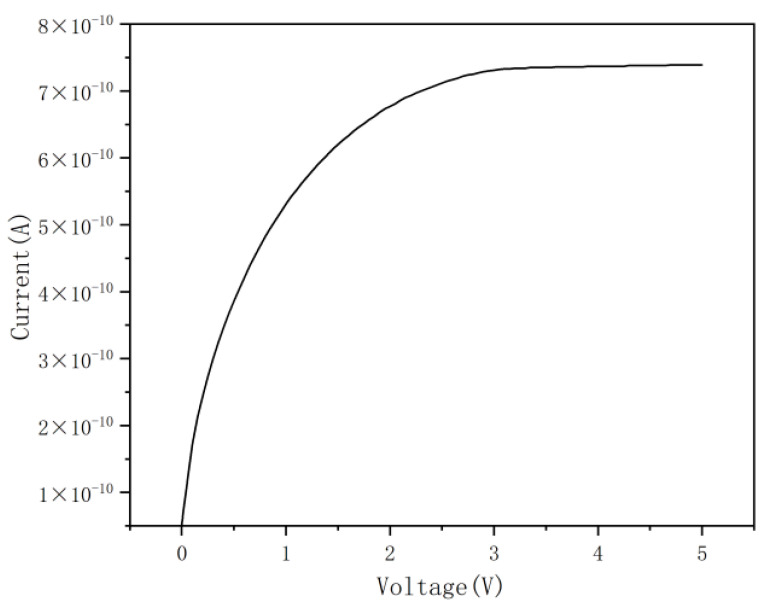
Leakage current curve of the DRIE Uneven-Etched 3D-Spherical electrode detector under various bias voltages.

Figure 10 depicts the leakage current curve of the DRIE Uneven-Etched 3D-Spherical electrode detector. The detector’s inherent noise is attributed to its leakage current, which depends on the internal structure of the device; consequently, reducing the leakage current enhances the detector’s sensitivity. As demonstrated, the leakage current in the DRIE Uneven-Etched 3D-Spherical electrode detector is very low, showing a considerable reduction (due to the reduction in effective unit cell volume) compared to conventional 3D-trench electrode Si detectors [15].

**Figure 11:**
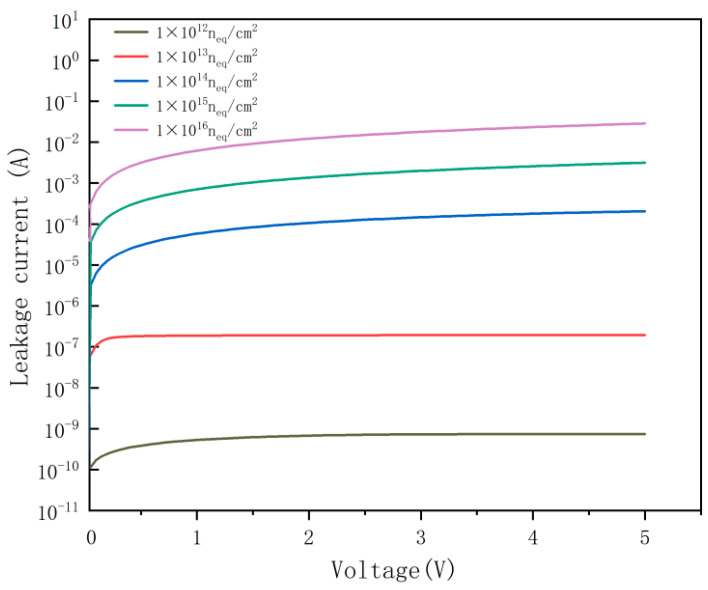
Leakage current of the DRIE Uneven-Etched 3D-Spherical electrode detector under the influence of the radiation fluence.

Figure 11 compares the leakage current of the DRIE Uneven-Etched 3D-Spherical electrode detector after radiation at various radiation fluences, indicating a growth in leakage current as the irradiation level increases. The following formula computes the detector’s leakage current [29]:

$$J=\frac{{\mathrm{qwAn}}_{i}}{2\tau }$$

Here, q is the elementary charge, n_i_ is the intrinsic carrier concentration in silicon, n_i_ = 1.5 × 10^10^/cm^3^, and τ is the minority carrier lifetime.

In a strong irradiation environment, the carrier lifetime will be shortened. The radiation-induced increase in leakage current per unit volume (J) is proportional to the radiation fluence. For 1 MeV equivalent neutron radiation fluence Φn_eq_, the change in current can be written as the following equation [30]:

$$\frac{J}{\mathrm{Vol}}=\alpha \Phi_{n_{\mathrm{eq}}}$$

where α = 4 × 10^−17^ A/cm is the damage coefficient, and Vol = wA is the volume of the depletion region [31,32].

From the above two equations, the value of τ is obtained as follows:

$$\tau =\frac{{\mathrm{en}}_{i}}{2\alpha \Phi_{n_{\mathrm{eq}}}}$$

By substituting this equation into the SRH recombination model of the simulation tool, the leakage current of the three-dimensional trench electrode detector after irradiation can be obtained in the simulation.

### 3.5. Capacitance of the DRIE Uneven-Etched 3D-Spherical Electrode Detector

A crucial parameter of a high-performance detector is its signal-to-noise ratio, defined as the ratio of useful signal to useless noise. The detection performance of the detector will be reduced greatly by the noise. Capacitance plays a key role in determining the noise level of the detector [13,33] and is determined by the detector’s collection electrode area. Our DRIE Uneven-Etched 3D-Spherical detector features a collection electrode that is significantly smaller than the conventional design, with an area that is only 5.44% of a full-cell covering electrode.

In the TCAD capacitance simulation, the following models are used:

(1) Physical Models: OldSlotboom bandgap narrowing, incomplete ionization, doping-dependent mobility, carrier scattering, high-field saturation (GradQuasiFermi), Lombardi vertical field mobility, SRH (DopingDep) recombination, Auger recombination, trap-assisted Auger recombination, and surface SRH recombination.
(2) Transport Model: Drift–diffusion model (Poisson equation and electron/hole continuity equations).
(3) AC Analysis Model: Small-signal linearization, frequency-domain admittance extraction, and single-frequency (1 MHz) capacitance calculation.
(4) Numerical Methods: Extrapolation, relative error control, undamped iteration, and quasi-static scanning (QuasiStationary).
(5) Solver: Blocked parallel direct sparse solver (Blocked+ParDiSo).

TCAD does not specify whether it includes parasitic contributions from floating rings, metal contacts, and neighboring cells in an array.

As the bias voltage V_bias_ rises, the depletion layer thickness w of the Si-PIN detector grows until full depletion is reached, where w equals d and stabilizes, no longer varying with further increases in V_bias_. As a result, once the detector is fully depleted, the junction capacitance C_d_ is proportional only to the electrode area for a specified detector thickness.

**Figure 12:**
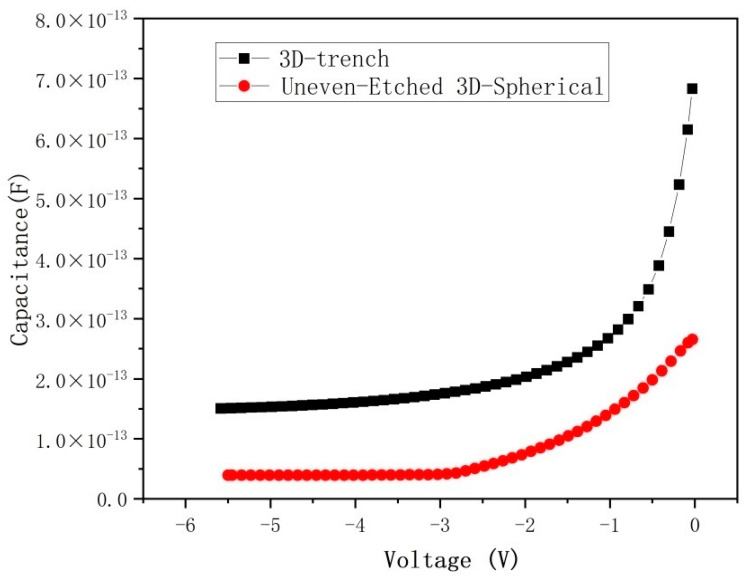
Capacitance curves for the DRIE Uneven-Etched 3D-Spherical electrode detector and traditional 3D-trench electrode silicon (R = 150 μm).

Figure 12 shows that the capacitance of the DRIE Uneven-Etched 3D-Spherical electrode detector is smaller than that of the traditional 3D-trench electrode. To obtain a fair comparison, we used the same detector parameters for both the 3D-Spherical electrode detector and the 3D-trench electrode shown in Table 1.

The simulated geometry capacitance of the 3D-Spherical electrode detector is around 4.1 × 10^−14^ F, while that for the 3D-trench electrode detector is 12.4 × 10^−14^ F. The DRIE Uneven-Etched 3D-Spherical electrode detector reduces the area of the detector electrode, which results in a reduced detector capacitance. Consequently, the signal-to-noise ratio and energy resolution of the detector can be improved. Compared to [17], our results are similar, but the detector’s fabrication method is not the same.

## 4. Electrical Characteristic Results with Different Structural Parameters (R and g)

### Full Depletion Voltage and Electron Concentration Distribution of the DRIE Uneven-Etched 3D-Spherical Electrode Detector with Different Structural Parameters

We obtain nearly the same electric field and electron concentration distributions as those shown in the previous section for various g values (R = 150 μm; g = 7 μm, 8 μm, 15 μm, 20 μm). According to the depletion voltage equation, the depletion voltage is solely determined by the distance between the cathode and the anode. The value of gaps between the adjacent circular trenches has little difference on the detector.

**Figure 13:**
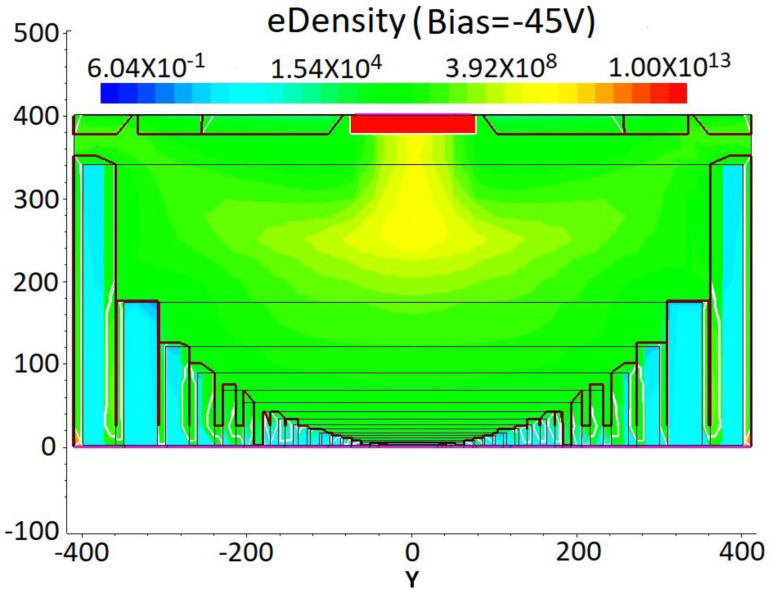
An electric concentration profile for the DRIE Uneven-Etched 3D-Spherical electrode detector under a bias voltage of −45 V. A schematic diagram of the electron concentration curves at a cut of Y = 0 in Figure 8 for various applied bias voltages is depicted in Figure 14a. At a bias voltage of −45 V, the detector becomes fully depleted of free electrons. In order to find the exact depletion voltage of the new detector, we simulated the electron concentration of the new detector at small stage steps between −40 and −45 V, with results shown in Figure 14b. Figure 14c is an enlarged schematic view of the yellow region in Figure 14b. The detector becomes fully depleted of free electrons when a bias voltage of −41 V is applied, which is taken as the detector being fully depleted. This value (−41 V) corresponds well to the theoretical value of −40.3 V from the depletion voltage equation.

We simulated an electron concentration profile of the DRIE Uneven-Etched 3D-Spherical electrode silicon detector with a much larger R value (R = 400 μm; g = 10 μm) for bias voltages of −10, −20, −30, −35, −40, and −45 V; the results with V = −45 V are shown in Figure 13, where full electron depletion can be seen.

**Figure 14:**
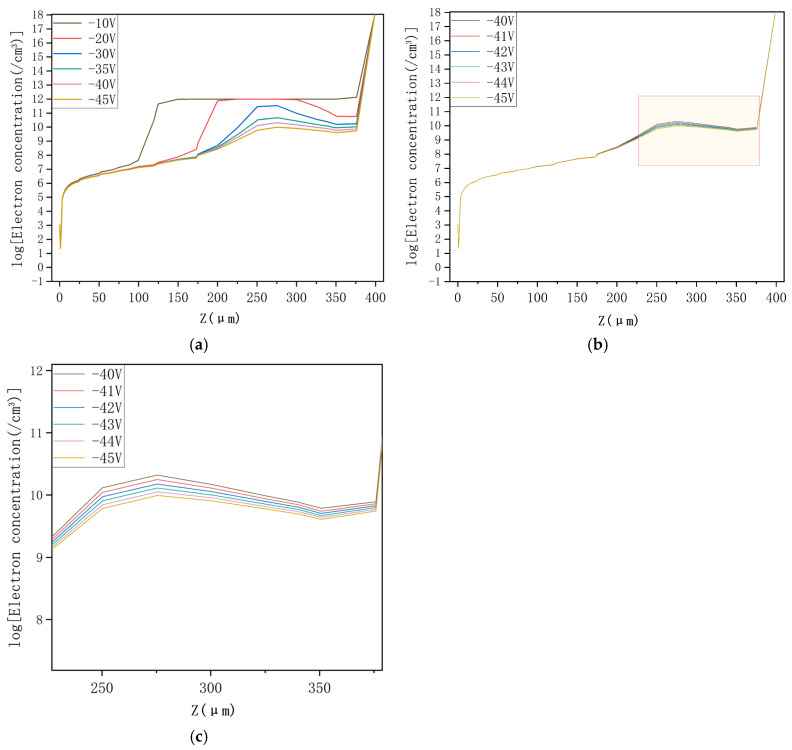
(**a**) Depth distribution of electron concentration in the innovative detector under various voltages. Magnified views of the electron concentration depth profiles of the innovative detector under various voltages (**b**) in this figure and (**c**) in Figure 14b.

## 5. Conclusions

In this work, a TCAD simulation study on the DRIE Uneven-Etched 3D-Spherical electrode detector was carried out. The distance from the cathode to the center anode of the DRIE Uneven-Etched 3D-Spherical electrode detector is the same at all angles; therefore, the new electrode detector has nearly no θ dependence in charge collection efficiency. We find that the DRIE Uneven-Etched 3D-Spherical electrode detectors, compared with the traditional 3D-trench electrode detector, have a much lower full depletion voltage. This is due to near-sphere-symmetry electrodes and electric field distribution with only radius R dependence in both strength and direction. The electric potential distribution, electric field distribution, and electron concentration distribution in our new detector are symmetrical. Electrical characteristics, such as leakage current, were also obtained. The leakage current of the detector is smaller, so the signal-to-noise ratio will be higher. The detector’s performance after irradiation was also simulated and analyzed. Compared with the traditional detector, the practical novel 3D-Spherical electrode detector has a lower capacitance. This would make it a good detector for application in photon science (e.g., X-rays) if we fabricate it using the DRIE etching method. This new design provides a practical method to realize a semi-spherical electrode with a single DRIE step, utilizing a special design. The theoretical research and findings related to these results also provide a solid foundation for the processing of the Uneven-Etched 3D-Spherical electrode using the DRIE etching method.

In summary, the structure design of the DRIE Uneven-Etched 3D-Spherical electrode detector, as well as that found in other studies [18,19], is a good basis for realizing a 3D-Spherical electrode detector. In future work, we will continue to study the properties of other 3D-Spherical electrode detectors and their arrays, as well as carry out practical detector fabrication.

## Figures and Tables

**Table 1:** Parameters of the 3D-Spherical electrode detector and the 3D-trench electrode.

	Neff (10^12^/cm^2^)	d (μm)	R (μm)	rc (μm)
3D-Trench	1.0	150	150	35
3D-Sphere	1.0	150	150	35

## Data Availability

The original contributions presented in this study are included in the article. Further inquiries can be directed to the corresponding author.
